# Fiddler crab electroretinograms reveal vast circadian shifts in visual sensitivity and temporal summation in dim light

**DOI:** 10.1242/jeb.243693

**Published:** 2022-03-09

**Authors:** Emelie A. Brodrick, Martin J. How, Jan M. Hemmi

**Affiliations:** 1 Living Systems Institute, University of Exeter, Exeter EX4 4QD, UK; 2 School of Biological Sciences, University of Bristol, Bristol BS8 1TQ, UK; 3 School of Biological Sciences & UWA Oceans Institute, University of Western Australia, Perth, WA 6009, Australia

**Keywords:** Gelasimus, ERG, Compound eyes, Light intensity, Dark adaptation

## Abstract

Many animals with compound eyes undergo major optical changes to adjust visual sensitivity from day to night, often under control of a circadian clock. In fiddler crabs, this presents most conspicuously in the huge volume increase of photopigment-packed rhabdoms and the widening of crystalline cone apertures at night. These changes are hypothesised to adjust the light flux to the photoreceptors and to alter optical sensitivity as the eye moves between light- and dark-adapted states. Here, we compared optical sensitivity in fiddler crab (*Gelasimus dampieri*) eyes during daytime and night via three electroretinogram (ERG) experiments performed on light- and dark-adapted crabs. (1) Light intensity required to elicit a threshold ERG response varied over six orders of magnitude, allowing more sensitive vision for discriminating small contrasts in dim light after dusk. During daytime, the eyes remained relatively insensitive, which would allow effective vision on bright mudflats, even after prolonged dark adaptation. (2) Flicker fusion frequency (FFF) experiments indicated that temporal summation is employed in dim light to increase light-gathering integration times and enhance visual sensitivity during both night and day. (3) ERG responses to flickering lights during 60 min of dark adaptation increased at a faster rate and to a greater extent after sunset compared with daytime. However, even brief, dim and intermittent light exposure strongly disrupted dark-adaptation processes. Together, these findings demonstrate effective light adaptation to optimise vision over the large range of light intensities that these animals experience.

## Abstract (precis)

**Summary:** Following descriptions of striking morphological changes in fiddler crab compound eyes from day to night, we report evidence for great changes in the associated visual sensitivity in *Gelasimus dampieri*.

## INTRODUCTION

Across the animal kingdom, different evolutionary directions, demands and limitations have created a huge diversity of eye designs. Often, as sensitivity in one aspect of vision is improved, physical constraints mean that another must be sacrificed. The fundamental limit is the number of photons that can be used for various tasks. For example, high spatial resolution can be achieved in a compound eye by packing more ommatidia into a smaller area to increase pixel density (Land and Nilsson, 2012; Horridge, 2005; Warrant, 2017). But this means that each facet lens must be smaller, letting in fewer photons and therefore lowering its absolute sensitivity, which is the minimum light intensity that can be detected by the eye. Absolute sensitivity is also limited by signal-to-noise ratios created by photon shot noise and the pathways of phototransduction (Field et al., 2005; Parag and Vinnicombe, 2017; Snyder et al., 1977). Contrast sensitivity will also be reduced as the eye struggles to collect enough light to discriminate two parts of a visual scene with different luminosity (O'Carroll and Wiederman, 2014; Pelli and Bex, 2013).

Very fast vision is useful for fast locomotion such as flight. An animal can speed up its temporal resolution, which is how quickly the visual system can respond to changes in luminosity over time (Eisen-Enosh et al., 2017; Warrant, 2017). This is achieved by shorter neural integration times for responding to light stimulation; however, it means a smaller number of photons are pooled per unit of time and, thus, the image will appear dimmer. As a consequence, animals that specialise in fast temporal resolution tend to have diurnal, terrestrial or shallow-water lifestyles where there is plenty of light (Gonzalez-Bellido et al., 2011; Miall, 1978; Theobald et al., 2007; Warrant, 1999; Laughlin and Weckström, 1993), while slower vision is typical of nocturnal animals (Aho et al., 1993; Fenk and Schmid, 2011). For an eye adapted to low light conditions, the goal is to maximise photon collection. So, it is common to find widened eye apertures in these animals, and their photoreceptors often pool the small amount of available light in space or time. Although this boosts absolute sensitivity and therefore contrast sensitivity in dim light, it results in poorer spatial or temporal resolution (Gonzalez-Bellido et al., 2011; Warrant, 2017, 1999; Stöckl et al., 2017). Therefore, visual systems tend to specialise in order to maximise sensitivity for different combinations of these tasks. Priorities can change over varying time scales, from months (seasonal or habitat changes) to hours (diurnal changes) to minutes or seconds (through movement driven changes such as animals flying between open and shaded areas or entering a dark refuge). Reversible and reactive changes in the eye help animals cope with these fluctuations in brightness (Land and Nilsson, 2012).

During daytime, the fiddler crab eye must prepare for exposure to the extremely bright light typically experienced on the surface of their semitropical or tropical mudflat habitats (Crane, 1975; Zeil and Hemmi, 2006). There is no previously published anatomical study on the eyes of our study species, *Gelasimus dampieri*, so we conducted a small examination ourselves to see whether they match trends found in other brachyuran crabs. Many crab species are known to undergo dramatic changes in optical structures from day to night (Leggett and Stavenga, 1981; Nässel and Waterman, 1979; Rosenberg and Langer, 2001), including the closely related fiddler crab *Afruca tangeri* (Brodrick et al., 2021). A study of the eye anatomy in *A. tangeri* revealed no evidence of pigment migration exists to help moderate light flux to the photoreceptors (Brodrick et al., 2021). However, their rhabdoms are narrow at midday in a light-adapted eye (∼2 µm diameter), with narrow lower crystalline cone tracts to match. These changes minimise acceptance angles and restrict the amount of light reaching the photoreceptors. At dusk, the volume of photopigment-packed microvillus membrane in the rhabdom increases five-fold to boost photon capture in dim light, taking up to 1 h to reach its maximum diameter (Stowe, 1983; Arikawa et al., 1987; Brodrick et al., 2021). This is accompanied by a widening crystalline cone tract, which theoretically doubles acceptance angles of the ommatidia at night after dark adaptation (Brodrick et al., 2021; Snyder, 1979). A larger diameter of the combined rhabdom and surrounding palisade also supports a larger number of wave-guide modes to propagate through (Land and Osorio, 1990; Stavenga, 2004). Using a formula proposed by Warrant and Nilsson (1998), these physiological changes would increase optical sensitivity to white light by 7.4 times in *A. tangeri* eyes (Brodrick et al., 2021), enabling nocturnal foraging and courtship behaviours. Several ghost crabs from the same Ocypodidae family of crabs undergo very similar changes in rhabdom volume (Rosenberg and Langer, 2001).

Here, we sought to assess how circadian alterations in eye anatomy and photoreceptive function affects the visual sensitivity of living *G. dampieri* fiddler crabs when light- and dark-adapted, via three electroretinogram (ERG) experiments. We assessed thresholds of light detection, temporal summation and rates of dark adaptation between day and night. The ERG recording electrode loop contacts the external surface of multiple facet lenses and a variety of underlying retinal cell types contribute to the signal, including slow responses of pigment cells and neurons of the lamina (Kohn and Minke, 2012), and so the combined synchronised depolarisation of many photoreceptor cell membranes in response to a flash of light is what primarily makes up an ERG signal. The amplitude of a photoreceptor response is proportional to the logarithm of the stimulus intensity (Kohn and Minke, 2012; Belusic, 2011).

Experiment 1 measured ERG responses to a series of 10 Hz flickering LED stimuli of increasing brightness. Responses were compared between light- and dark-adapted eyes during daytime and night, producing four treatments. We predicted dark-adapted eyes to have lower response thresholds than light-adapted eyes. From electron microscopy study of ocypodid and other brachyuran crabs, the rhabdom and crystalline cone changes associated with dark adaptation at night are largely inhibited during daylight hours (Brodrick et al., 2021; Rosenberg and Langer, 2001). Therefore, we expected dark-adapted eyes to be able to detect dimmer stimuli at night than during the day.

Experiment 2 assessed whether neural temporal summation was activated in dim light to increase integration times and photon capture. We compared the crabs' flicker fusion frequency (FFF) after light and dark adaptation during daytime and night (as in experiment 1). Crabs were presented with a stimulus series with different flicker frequencies to which the ERG response amplitudes provide a direct measure of the crab's ability to resolve the flicker in time. We expected FFF to decrease when dark-adapted as a result of temporal summation owing to an expected slower response profile of the visual system, which is necessary for temporal integration (Stöckl et al., 2016). The 100 Hz low pass setting of the amplifier slightly reduces response amplitudes to high frequency stimulation (by approximately 30% for a 100 Hz stimulus compared with a 1 Hz stimulus; WPI DAM50 Instruction Manual). However, this is negligible compared with the 100-fold change in measured response amplitude (see Results). We interpreted the data in a relative fashion, so our conclusions are not affected by this amplifier setting.

Experiment 3 explored the speed at which fiddler crabs can dark-adapt during the day or night. We recorded ERG responses to identical stimulus presentations of unchanging intensity at regular intervals as the fully light-adapted eye adjusted to the dark over a 1-h period. Tests were conducted during daytime and at night (>2 h after sunset) to compare the rate and extent of sensitivity changes as the eye dark-adapts. In the fiddler crab *A. tangeri*, the dramatic rhabdom widening process around sunset involves elongations of many thousands of microvilli in each ommatidium, in addition to widening of the crystalline cone aperture to allow more light to enter each ommatidium (Brodrick et al., 2021). This process must require a significant energetic investment and is unlikely to happen quickly or be readily reversible (rhabdoms take ∼1 h to widen fully), making it an unsuitable strategy to moderate short periods of brightness fluctuation. Therefore, after dusk we expected a steep initial increase in sensitivity owing to aperture widening and/or temporal summation, followed by a more gradual increase in sensitivity over the hour as the rhabdom widens. However, during daytime, we expected a smaller initial increase in sensitivity (from temporal summation only), with eyes ceasing to undergo further anatomical adaptation (Brodrick et al., 2021). This would mean that maximum sensitivity might be reached sooner during daylight hours. And without rhabdom widening, the eyes were predicted to be much less sensitive after 60 min at this time, compared with at night.

## MATERIALS AND METHODS

### Animal care and preparation

Collections of fiddler crabs of the species *Gelasimus dampieri* (Crane 1975) were made from tidal mangroves near Learmouth, Western Australia (22°18′01.0″S, 114°09′11.3″E), and transported to a large artificial mudflat at the University of Western Australia, Perth. This artificial mudflat included a ‘tidal’ immersion/emersion cycle and a 12 h:12 h light:dark cycle (changes at 06:00 and 18:00 h) to emulate natural conditions. Before experiments, individuals were captured from the artificial mudflat system and held in a smaller facility with equivalent light and tidal conditions. Crabs were fed dried flake food (Aqua One Tropical Flakes) every 2 days. Eight individuals (four males, four females) participated in all three ERG experiments.

Crabs were pre-adapted to either bright light or darkness and experiments were performed during day and/or at night, avoiding the time 2 h before and after sunset. To light-adapt, crabs spent 2 h inside an aluminium foil-lined container with 1–2 cm seawater illuminated from above by a large ring of white LEDs behind layers of light-diffusing film (no. 216, Lee Filters, Andover, UK) with intensity between 24.1 and 40.3 µW cm^−2^ (at crab eye level, depending on viewing angle) and identical spectral shape to the ERG stimulus. Alternatively, they were placed in a light-proof box inside a darkroom at sunset to dark-adapt, where they remained until >2 h after sunset or until the following day. Whilst preparing the dark-adapted crabs for experiments, their eyes were not exposed to any light except a dim red lamp with emission >620 nm, which is visible to humans but barely detectable to the *G. dampieri* visual system (Jessop et al., 2020). Preparation and maintenance of eyes in a light-adapted state was assisted by a bright white diffused LED array in front of the animal to illuminate the part of the eye we stimulated during experiments.

Crabs had a small, insulated plastic disk adhered to their carapace with cyano-acrylate glue, which remained in place until their next moult. The disk was used to temporarily glue the crab to a mounting post during experiments. Claws were restrained with electrical tape to avoid dislodging the electrode during the experiment. With a small dab of cyano-acrylate glue halfway up the back of an eyestalk, one eye was temporarily fixed still to a wooden post in its natural upright position. To prevent gill desiccation during experiments, crabs were suspended from the mounting post, half immersed in a tank of seawater. Crabs were carefully removed unharmed from the apparatus between experiments.

### Ethical standards

All animals were treated according to UWA Animal Ethics Committee (AEC) approved methods (UWA AEC project numbers RA/3/100/1515 and RA/3/400/1020).

### ERG apparatus

ERG recordings took place inside a light-proof, grounded Faraday cage on a pressurised anti-vibration table. The recording signal electrode, a 254 µm diameter platinum wire shaped into a small loop and coated with conductive gel (Livingstone International Pty Ltd, NSW, Australia), was put in gentle contact with the corneal surface on the lateral region of the upright eye. This allowed the frontal eye an unobstructed view of the stimulus. An indifferent reference electrode, a pellet-shaped silver–silver chloride wire inside a light-proof rubber shield, was placed inside the seawater bath next to the crab. The ERG signal was amplified 1000 times using an AC differential amplifier (DAM-50, World Precision Instruments, Sarasota, FL, USA), with a bandpass filter set to 1–100 Hz. The signal was visualised on a digital storage oscilloscope (2211, Tektronix, OR, USA) and digitised by a multi-function data acquisition unit (USB-6353 X-series, National Instruments, Austin, TX, USA), sampling at 5 kHz. Custom MATLAB programs (R2015b, MathWorks, Natick, MA, USA) were used to acquire and analyse the signal and control the stimulus.

### Light stimulation

**Fig. 1.**
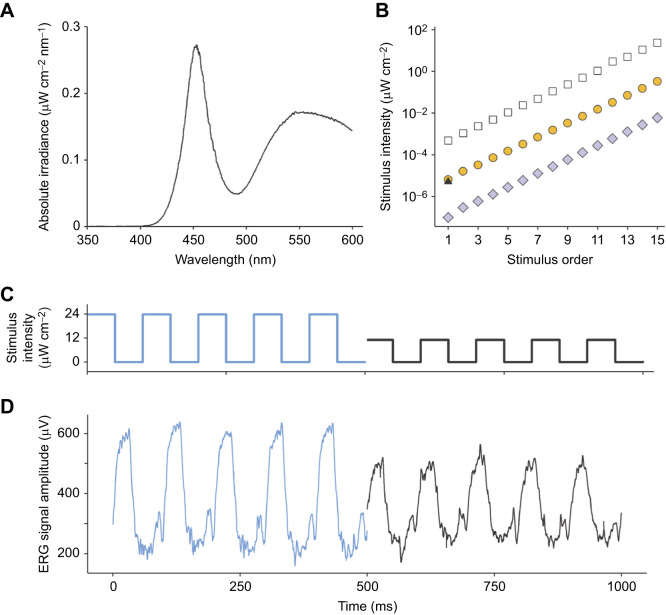
**Experimental light stimulation and typical ERG response examples.** (A) Spectrum and intensity of light stimulation. Absolute irradiance spectrum of white LED stimulus used in ERG experiments set to maximum brightness, without neutral density filters. A brighter LED array with identical spectrum was used to light-adapt crabs before experiments. (B) Intensity of the 15 experimental stimuli used in ERG experiments on a log_10_ scale. White squares represent stimuli used for light-adapted treatments during both day and night. Dark-adapted treatments were shown dimmer stimulus ranges created using a neutral density filter, two layers during daytime (yellow circles) and four at night (lilac diamonds). Stimuli were shown in stepwise order of dimmest to brightest. The black triangle (at *x*=1) indicates the stimulus intensity used repeatedly in experiment 3. (C) The 10 Hz square wave flicker of the brightest stimulus light intensity presented (in experiment 1) is plotted in blue, followed by a stimulus with half the intensity, black, above the corresponding ERG signal traces (D) from the same fiddler crab's eye.

The light from a ring of five white LEDs (5 mm, C503D-WAN, Cree, USA) was projected through a 1 mm thick, 3 cm diameter Teflon diffuser, producing an even, diffuse illumination circle (spectrum in Fig. 1A). The front of the recording eye was positioned 3 cm away, in line with the stimulus centre. The stimulus subtended a 53 deg visual angle as viewed from the crab eye. No other light source was present within the Faraday cage. The LED stimulus was controlled via MATLAB and a custom-built RGB LED controller, which moderated intensity and flicker frequency as per the experimental requirements. The LEDs could be dimmed linearly through 49,000 steps via pulse-width modulation with an underlying flicker rate of 1 kHz, which is invisible to the animals. To lower the stimulus intensity further, layers of 0.9 neutral-density filters (no. 211, Lee Filters) were slotted into the tube in front of the LEDs when required. The absolute irradiance spectrum of each different stimulus intensity used in the experiments was measured with a calibrated spectrometer (USB-2000+, Ocean Optics, Largo, FL, USA) and 600 nm diameter optical fibre.

Total irradiance (µW cm^−2^) for each stimulus intensity was determined by calculating the area beneath the spectrum curve within the wavelength range 300 to 600 nm, for which the *G. dampieri* visual system is sensitive (Jessop et al., 2020). It is worth noting that the ERG stimulation lacked ultraviolet wavelengths (<400 nm), which would be present in sunlight and are thought to be detected by the crab's R8 photoreceptors (Jordão et al., 2007; Horch et al., 2002).

Each stimulation lasted 21 s, which provided reliable signal averages and allowed us to check for any changes to the animal's adaptation state during recordings. During intervals between each presentation, a light-adapted state was maintained (when required) by setting the stimulus LED to full intensity (no flicker). Alternatively, dark-adapted state was maintained with the stimulus off in between recording periods. Preliminary trials indicated that 60 s inter-stimulus intervals were sufficient to maintain adaptation states. In the experiment that investigated the rates of dark adaptation, we used inter-stimulus intervals of 4 min.

### Experiment 1: intensity thresholds

Intensity thresholds were measured under four different treatment conditions: light-adapted during the day and at night, and dark-adapted in the day and at night. Each individual crab was tested in all four conditions. In order to measure the crabs' sensitivity thresholds, each crab was presented with an increasing intensity series of 15 stimuli, with one shown every 60 s. Each stimulus consisted of a 21 s, 10 Hz on–off square wave flicker (see examples in Fig. 1C). The stimulus intensity ranges were selected to cross the absolute detection thresholds of the fiddler crabs in each condition, established via preliminary trials where they were found to vary between treatments. The intensity of consecutive stimuli increased by one-third of a log unit each time (values in Fig. 1B). With decreasing stimulus intensity, the ERG response at the stimulus frequency diminishes in amplitude until it is no longer above the noise level. For two different light intensities, the first twice as bright as the second (Fig. 1C), the resulting ERG response traces from an example crab's eye (Fig. 1D) allow visualisation of a typical signal.

ERG response amplitudes were acquired using a custom MATLAB program (J. M. Hemmi). Response amplitudes were fitted with a best-fit line and the intensity required to evoke a threshold mean response amplitude of 1 μV was estimated for each individual in all four conditions. This was done in order to eliminate the effects of small spurious electrical signal noise generated by the LEDs. This procedure slightly underestimates absolute sensitivity thresholds but allows us to compare relative light intensity between treatments.

### Experiment 2: temporal resolution

An experiment measuring the FFF to a standardised light stimulus followed shortly after experiment 1. In between the experiments, the light stimulus was either left on full brightness (light-adapted crabs) or turned off (dark-adapted crabs) to maintain the crabs' adaptation state. To compare FFF thresholds at different adaptation states, we calculated for each animal in their current treatment the light intensity that provoked a response amplitude of 3.2 μV, which was estimated based on the least-square fit of the data from experiment 1. This response threshold was chosen as it was reliably above noise but did not lead to light-adaptation changes in dark-adapted eyes. Although intensity differences were bound to affect response amplitudes, we chose to normalise the stimulus intensity to the individual's own recent response curves to provide truer comparisons for individuals between treatments. In our trial experiments, using fixed intensity values meant that crabs with the highest or lowest sensitivities in experiment 1 were producing anomalous results in experiment 2. Using the individually determined stimulus intensity, a series of 11 stimuli lasting 21 s each were presented to the crab, with an inter-trial interval of 60 s. The square wave flicker frequency was increased from 10 to 120 Hz in stepwise increments of 10 Hz. Data for 50 Hz were excluded from the results owing to mains frequency contamination of the signal. Between presentations, the adaptation state was maintained as outlined above. The FFF was deemed the lowest frequency at which the crab produced a significant response above a threshold of 0.6 μV.

### Experiment 3: rates of dark adaptation

Experiment 3 aimed to measure the speed of dark adaptation at different times of the day. All animals were fully light-adapted (as described in experiment 1), then the adaptation lights were switched off at the start (at 0 min) to begin the experiment. The crabs remained in darkness in the Faraday cage for 1 h to encourage the eye to dark-adapt. Every 4 min, the sensitivity of the eye was tested by recording the ERG amplitude to the same dim stimulus: a 10 Hz square wave on–off flicker with ‘on’ intensity of 3.84×10^−4^ µW cm^−2^ (black point in Fig. 1B). This intensity was chosen as it was relatively dim and usually failed to provoke a significant response when crabs were fully light-adapted, while provoking reliable, significant responses in dark-adapted crabs. All eight crabs were tested both during daytime (>2 h before sunset) and at night (2–5 h after sunset), in a random order balanced across crabs. To test whether the stimulation affected the time course of the crabs' dark adaptation process, each crab was tested again on a different day; however, only the first and last stimulus presentations were shown, at 0 and 60 min, allowing an hour of uninterrupted dark adaptation in between.

### Data processing

A median filter with 11 ms range was applied to the raw data to remove frequency artefacts in the signal, e.g. the crab heartbeat, and anomalies of 5 standard deviations or more from mean signal amplitude were also removed; these occurred occasionally when the crab's legs or claws moved during the recording. From the remaining signal, mean response amplitude over the 21 s recording was calculated, along with mean, maximum and minimum noise values. To do so, fast-Fourier transforms (FFTs) were applied to the ERG signal recordings to separate their constituent frequencies and the amplitude of the response at the stimulation frequency was calculated. Probability (alpha) values were also calculated for each signal recording to determine whether the response was statistically different (at the 5% level) from the noise level. ERG response thresholds of 1 μV (experiment 1) and 0.6 μV (experiment 2) were used to compare between treatment groups as they were reliably above noise levels in the signal for all animals. The slightly higher comparison threshold in experiment 1 was used to eliminate unreliable low values around the crabs' absolute visual sensitivity threshold.

### Statistical analyses

Using the R software package ‘lme4’ (Bates et al., 2014) (v3.5.1; http://www.R-project.org), a linear mixed effects model (LMER) was fitted to data for both ERG experiments, using response amplitude as the continuous fixed effect each time. Crab identity was given as a random effect. Using the R package ‘predictmeans’ (v1.0.1; https://CRAN.R-project.org/package=predictmeans), several other potential explanatory fixed effect terms (see Results) were tested for statistical significance by running 10,000 random permutation recalculation tests (Lee and Braun, 2012) on the data. Non-significant variables were excluded to produce a final working model.

For experiments 1 and 2, *post hoc* pairwise comparisons between each of the four conditions were performed with Tukey contrasts using the glht function in R package ‘multcomp’ (Hothorn et al., 2008). For experiment 3, ERG responses at 60 min for the control test (uninterrupted dark-adaptation) were compared with the ERG responses at 60 min in the main test (when stimuli were presented every 4 min). Welch's *t*-tests were used for analyses of linear data because they had non-homogeneous variances (Bartlett's test) and residuals were not normally distributed (Shapiro–Wilk test).

### Light and electron microscopy

Two days after completion of the ERG experiments, the eyes of four of the participant crabs used in those experiments were fixed for histological examinations. Two crabs were light-adapted in the same manner as for ERG experiments and eyes were dissected in bright light, one individual at noon and the other at midnight. Meanwhile, two crabs were dark-adapted and their eyes dissected in a dark room using dim red light at noon or midnight. Eyes were stored in phosphate buffered 2.5% glutaraldehyde and 4% paraformaldehyde fixative overnight at 4°C. They were washed and transferred to phosphate buffer before being transported to the UK to undergo sample preparation and embedding in EPON resin (see method in Brodrick et al., 2021 for detail). The frontal facing eye equator region of embedded eyes was cut using a ultramicrotome (EM-UC6, Leica, Wetzlar, Germany) into thick (1000 nm) sections for light microscopy (Leica DM750) and semi-thin (70 nm) sections for electron microscopy (Tecnai 12, 120 kV BioTwin Spirit, FEI Company, Hillsborough, FL, USA). Observations were made on the relative cross-sectional areas of crystalline cones and rhabdoms and these were compared, conjecturally, to similar data on *A. tangeri*.

## RESULTS

### Intensity thresholds

**Fig. 2.**
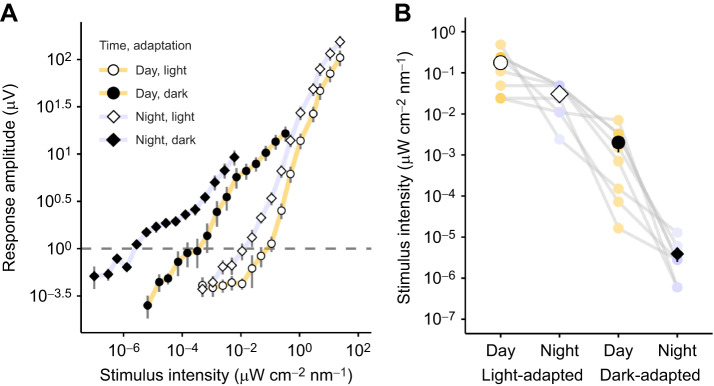
**Experiment 1: ERG responses vary according to the time of day and the animals' adaptation state.** (A) Mean ERG response amplitudes (error bars show s.e.m.) from *Gelasimus dampieri* (*n*=8) to increasing stimulus intensities recorded in a light-adapted state (open points) or a dark-adapted state (filled points), during daytime (circles) or night (diamonds). A response threshold of 1 μV is marked by a dashed line at which ERG amplitudes were reliably above noise in the signal, allowing comparison between treatments. (B) The stimulus intensity required to elicit a threshold response amplitude of 1 μV is plotted for each crab (lilac and yellow points for individuals connected with grey lines). Black or white points represent the mean±s.e.m. bars for the four conditions.

Increasing the stimulus intensity caused a corresponding increase in the ERG response of the eye (χ^2^_1_=730.3, *P*<0.001, *n*=8) and there were large differences between all treatments (Table 1, Fig. 2A,B). The interaction between adaptation state and time of day had a significant effect on the ERG response amplitudes (χ^2^_1_=51.7, *P*<0.001). When the animals were light-adapted during daytime, the mean stimulus intensity required to provoke a threshold response of 1 μV was significantly higher by a factor of 5.8 than when light adaptation was prolonged during the night (see Table 1 for all mean values and statistical test results). Dark-adapted eyes gave larger response amplitudes than when light-adapted, during both day and night. Dark-adapted eyes were especially sensitive at night, when the stimulus to provoke this response threshold was three orders of magnitude dimmer than in the day.

**Table 1.**
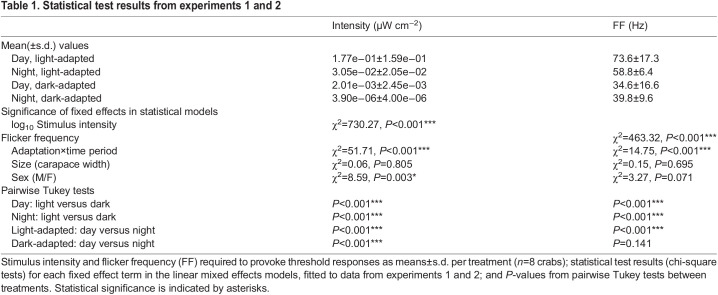
Statistical test results from experiments 1 and 2

Sex of the crab also significantly correlated with response amplitude. Females had higher response amplitudes than males (χ^2^_1_=8.6, *P*=0.003). There was no effect of crab size (carapace width, χ^2^_1_=0.06, *P*=0.805).

### Temporal resolution

**Fig. 3.**
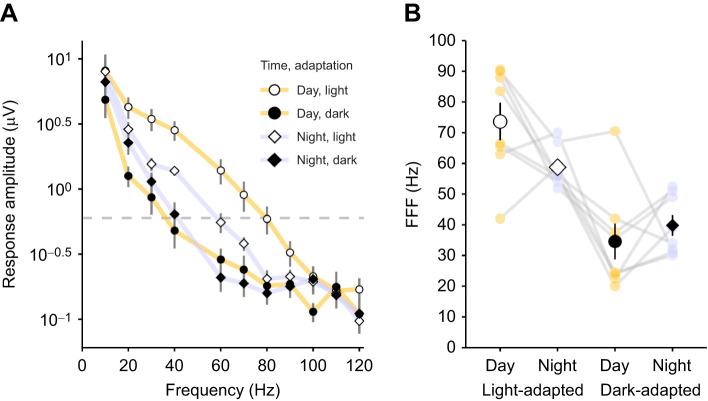
**Flicker fusion frequency (FFF) for a standardised stimulus intensity is lower when crabs are dark-adapted.** (A) Mean ERG response amplitude with increasing stimulus flicker frequency for *G. dampieri* (*n*=8) tested when light-adapted (open points) and dark-adapted (filled points) in daytime (circles) and night (diamonds). Error bars show s.e.m. A horizontal dashed line marks the 0.6 μV critical threshold used to plot values in B. Note, stimulus intensity for each individual was selected from the least-square fit of data from experiment 1, using estimations of intensity required to provoke a 3.2 μV response in the eye. Consequently, stimulation intensity varied between treatments and animals. (B) The flicker frequency for individual crabs (lilac and yellow points for individuals connected with grey lines) required to provoke a threshold response of 0.6 μV (dashed line in A). Black or white points represent the mean value for the four conditions, error bars show the s.e.m.

In all four treatments the ERG response amplitudes showed a negative association with increasing stimulus flicker frequency (χ^2^_1_=463.3, *P*<0.001, *n*=8) (Fig. 3A). There was a significant interaction between adaptation state and time of day on response amplitudes (χ^2^_1_=14.8, *P*<0.001). Light-adapted crabs produced larger responses than dark-adapted crabs for frequencies up to 90 Hz, particularly during the day. The FFF required to produce a 0.6 μV response (Fig. 3A, dotted line) was significantly higher during daytime (mean±s.e.m.=73.6±6.1 Hz) than at night (58.8±2.3 Hz) for light-adapted animals. When the crabs were dark-adapted, temporal resolution was lower still, but FFF was not significantly different between day (34.5±5.9 Hz) and night (39.8±3.4 Hz). Carapace width (χ^2^_1_=0.2, *P*=0.695) and sex of the crabs (χ^2^_1_=3.3, *P*=0.071) had no significant effect on the responses.

### Rates of dark adaptation

**Fig. 4.**
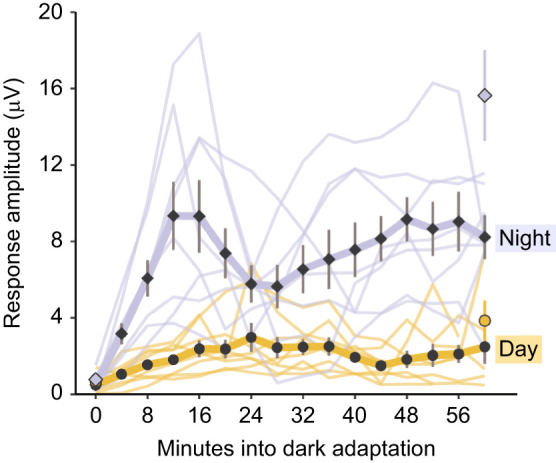
**Experiment 3: Rates of dark adaptation during day and night.** Response amplitude to a 10 Hz flickering stimulus of 3.84×10^−4^ µW cm^−2^ intensity, over the course of 1 h, shown in 4-min intervals as *G. dampieri* (*n*=8) adapt from bright light to darkness. Data for each individual are shown as thin yellow and lilac lines for tests carried out during the day or night, respectively. Black filled points and vertical lines show mean response amplitudes for day (circles) and night (diamonds) recordings. Points filled with yellow or lilac at 0 and 60 min show the mean response amplitudes. Error bars show the s.e.m. for the same eight crabs when they were tested on a different day at just these two times, allowing an hour of uninterrupted dark adaptation in between.

ERG response amplitudes increased with time during dark adaptation (χ^2^_1_=41.8, *P*<0.001, *n*=8) and this occurred faster and to a greater extent at night than in the day (χ^2^_1_=118.9, *P*<0.001) (Fig. 4). Carapace width (χ^2^_1_=0.09, *P*=0.768) and sex of the crabs (χ^2^_1_=0.02, *P*=0.879) did not significantly affect ERG responses. When shown the first stimulus (0 min), fully light-adapted eyes gave similar responses in daytime and at night (Welch’s *t*-test, *t*_14.0_=−0.09, *P*=0.933).

The adaptation time course could be broadly separated into three periods. The slope of increase in response amplitude during the first 12 min of dark adaptation was significantly steeper after sunset compared with during daylight hours (χ^2^_1_=32.8, *P*<0.001), when responses increased more slowly and to a lesser extent. In the second period, between 12 and 24 min, mean response amplitude continued to increase during daylight hours (Welch *t*-test, *t*_9.5_=3.3, *P*=0.008). However, a surprising decrease in response amplitude was measured in all crabs at night during this 12–24 min period (Welch’s *t*-test, *t*_13.2_=−2.2, *P*=0.042). In some individuals this was extreme, sometimes almost reaching the initial response level of the light-adapted state (finer lilac lines, Fig. 4). The third period, after 26–28 min, individual response amplitudes started to climb again, before some peaked and declined again towards the end of the hour. The mean final (60 min) response amplitude at night (8.2 μV) was higher than during daytime (2.5 μV) (Welch’s *t*-test, *t*_8.9_=−5.2, *P*<0.001).

Response amplitudes for the control test (no testing stimuli during dark adaptation) at night were significantly higher (mean value 15.6 μV) than the final values for when the stimulus was presented throughout the hour (mean response 8.2 μV) (Welch’s *t*-test, *t*_9.4_=2.9, *P*=0.018). This difference indicates that the stimulus itself had a strong effect on the adaptation of the eye and resulting response amplitudes, despite its relatively dim intensity and brief, intermittent presentation. During the day, when there were smaller overall increases in response amplitudes, some individuals also produced a peak and decline curve in response amplitude during the 1 h test period, with a similar shape but less pronounced than at night. By 60 min, a mean response amplitude of 2.5 μV was recorded from the crabs during daylight hours. This was slightly lower, but not significantly different to the control treatment at 60 min, when dark adaptation was uninterrupted by stimulus presentations, with a mean response of 3.8 μV (Welch’s *t*-test, *t*_12.9_=−0.9, *P*=0.344).

### *Gelasimus dampieri* eye anatomy

**Fig. 5.**
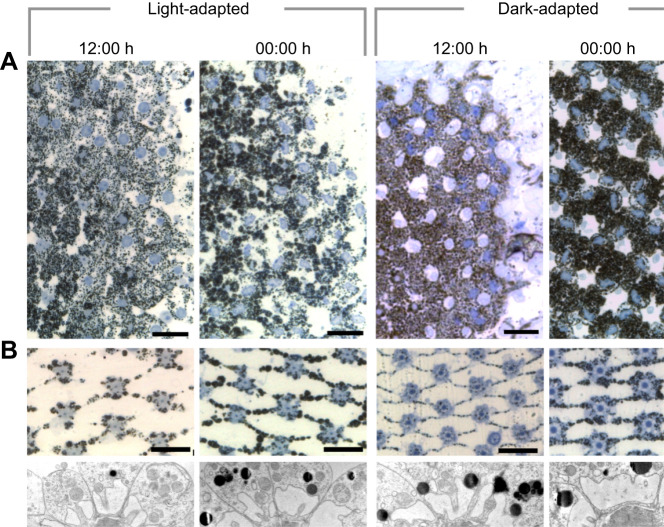
**Histological examples from *G. dampieri* eye samples.** (A) Light micrographs of oblique sections (1 µm thick) through the primary pigment cell layer of four eyes fixed in different adaptation states. Towards the right side of each panel are the distal ends of the primary pigment cell bodies containing their nucleus (stained bright blue). Scale bars=20 µm. (B) Several R8 cells in cross-section showing differing rhabdom diameters. The ommatidia are screened from one another by a network of thin primary pigment cell projections. Note the density of pigmentation in the cells between night and day. Scale bars=20 µm. (C) Rhabdoms in cross-section imaged with TEM. Scale bars=2 µm.

Presented in Fig. 5 are eyes from four individual crabs, each fixed in one of the four adaptation states used in our ERG experiments. Although the small sample size (*n*=1) does not permit statistical comparisons or strong conclusions, the observations and measurements we took from the histological sections provide some supporting evidence for our hypothesis that their eyes undergo similar light-adaptation mechanisms to that of another known fiddler crab, *A. tangeri* (Brodrick et al., 2021). Pigment cells are located at the base of crystalline cones where they meet the rhabdom. The distal parts of these cells are sparsely pigmented in light-adapted eyes but dense in dark-adapted eyes, potentially suggesting some additional pigment migration (Fig. 5A). The rhabdoms are wider in the dark-adapted crab that was fixed at midnight than in the other treatments (Fig. 5B,C).

## DISCUSSION

### Circadian changes in visual sensitivity

ERG responses revealed vast differences in optical sensitivity in light- and dark-adapted fiddler crabs between day and night. The stimulus intensities needed to provoke the same threshold response in the crabs varied greatly, over six orders of magnitude. With both anatomical changes (rhabdom and crystalline cone diameters) and physiological alterations (temporal summation), fiddler crab apposition eyes can effectively increase visual sensitivity for dim light vision faster and to a greater extent after sunset than during hours of daylight. This suggests that they are well adapted for activity on the mudflat surface, not only during bright daylight conditions, but also at dusk and possibly at night.

During daytime, the *G. dampieri* visual system needs to cope with the extremely bright conditions on the exposed tropical marine flats of northern Australia (Crane, 1975; Zeil and Hemmi, 2006). Assuming that this species employs similar light adaptation mechanisms to *A. tangeri* fiddler crabs (Fig. 5) (Brodrick et al., 2021)*,* narrow acceptance angles and fine rhabdoms in light-adapted eyes let reduced amounts of light reach the photoreceptors. In addition to short neural integration times, these would effectively screen out excess bright light typical in their habitat at this time, preventing overstimulation or photodamage to allow optimal contrast sensitivity (Brodrick et al., 2021; Zeil and Hemmi, 2006; Cronin and Forward, 1988). This would explain why a relatively bright stimulus was required to produce a threshold ERG response amplitude in experiment 1 when light-adapted during daytime. In contrast, dark-adapted crabs at night produced threshold responses to light intensities that were on average 45,000 times dimmer. This massive boost in visual sensitivity can be attributed to temporal summation in response to dim light, in addition to widened crystalline cones and rhabdoms (Brodrick et al., 2021; Rosenberg and Langer, 2001), which allow greater rates of photon capture. This can allow fiddler crabs to safely forage and interact when the lack of available light would vastly reduce the contrast of objects such as potential predators against the dark sky (O'Carroll and Wiederman, 2014). Close to dusk, spectral sensitivity in *G. dampieri* also shifts 25 nm towards longer wavelengths (Jessop et al., 2020), which is also likely to be associated with dark-adaptation processes. This may also have caused a slight change in overall sensitivity to our white LED stimulus owing to the characteristic shape of the emission spectrum (Fig. 1A).

Testing the crabs in a dark-adapted state during daylight hours allowed us to investigate whether differences in optical sensitivity are a result of light exposure only, or whether intrinsic circadian clocks also regulate optical sensitivity in fiddler crabs. Anatomical studies of other intertidal crab species (Leggett and Stavenga, 1981; Nässel and Waterman, 1979; Rosenberg and Langer, 2001; Stowe, 1981) have consistently revealed that rhabdoms and apertures do not reach their full size during long periods of darkness in day. We know that neural integration times lengthen to increase optical sensitivity for dim light vision regardless of time (experiment 2). However, the reduced ERG responses from fiddler crabs in experiment 1 during the day compared with at night also suggest that the eye structures were at least partially in light-adapted anatomical state at this time. This is corroborated by our histological examples of *G. dampieri* rhabdoms (Fig. 5). Because we did not expose the dark-adapted crabs to any light since the previous dusk, some light adaptation (rhabdom narrowing) must have occurred in the morning to explain the reduced sensitivity during the day, relative to at night. In histological studies of other laboratory-housed crab species (*Hemigrapsus* and *Leptograpsus*), their rhabdoms began to narrow in darkness just before or after dawn, when their artificial lighting is usually switched on (Arikawa et al., 1987; Stowe, 1983). These findings strongly indicate circadian clock involvement in the anatomical light-adaptation process. Light exposure also appears to be necessary to complete the light-adaptation process because our previous study on *A. tangeri* fiddler crabs showed that, in the absence of light, rhabdoms were only partially degraded by midday (Brodrick et al., 2021).

Bright light exposure is an important regulator of adaptation state. It triggers rhabdom degradation regardless of time and increases temporal resolution. Circadian clocks also predict the adaptation state between day and night, inhibiting the relatively slow processes of dark adaptation from happening during daylight hours to protect and optimise the eye. Sudden bright light exposure would temporarily oversaturate (and blind) a very sensitive fiddler crab visual system, leaving it vulnerable to predators on the mudflat. Maintaining a less sensitive, mostly light-adapted state throughout the day would prove beneficial in protecting the eye from damaging radiation or photopigment bleaching when it emerges onto the bright mudflat surface after a long period inside its burrow during daytime (Leggett and Stavenga, 1981).

### Fiddler crab vision is fast during day

During the daytime, mean FFF in light-adapted eyes was 74 Hz and two individuals in this treatment produced threshold responses close to 90 Hz. This indicates that very fast vision is possible in fiddler crabs, and brighter light stimulation (e.g. provided by strong sunlight) may facilitate even faster temporal resolution by providing a higher signal-to-noise ratio (Lisney et al., 2011; Laughlin and Weckström, 1993; Meyer-Rochow and Lindström, 1997). Temporal resolution exceeding 70 Hz is fast for a crustacean (Caves et al., 2016; Cohen and Frank, 2006; Doujak, 1985; Frank, 1999, 2000; Meyer-Rochow, 2001) and could be linked to lifestyle and predation pressures in a primarily terrestrial environment. Fast vision is often correlated with quick locomotion (Laughlin and Weckström, 1993; O'Carroll and Wiederman, 2014), and air provides a less dense and resistant medium for the relatively non-streamlined crab body plan, allowing more rapid movement than in water (Martinez, 2001). However, the strongest driver for fast vision is likely to be the birds that attack the crabs. The avian predators are often distant, fast-flying and in regular pursuit (Hemmi, 2005; Smolka et al., 2011; Land, 1999), and rapid vision may also be beneficial in tracking flight trajectories of nearby birds, or to assess the threat level. Visual flicker appears to be an important surveillance cue to fiddler crabs, and is likely to aid in identification of flapping bird wings at distance, alerting them early to danger (Smolka et al., 2012; Hemmi, 2005; Smolka et al., 2011). To best identify distant bird predators from other objects using these rapid flickering movements with fairly poor spatial acuity (Bagheri et al., 2019; Smolka and Hemmi, 2009), fiddler crabs must rely on relatively fast vision. It is possible that they use information on the strength of the flicker to assess the flight speed of animals above them (Hemmi, 2005; Hemmi and Tomsic, 2012).

### Temporal summation enhances dim light vision

Anatomical changes may be chiefly responsible for the differences in relative sensitivity and contrast sensitivity measured in *G. dampieri*. However, experiment 2 provides a convincing demonstration that temporal summation is an additional strategy used by this species to improve sensitivity in dim light. This is shown by the strong difference in FFF between dark- and light-adapted animals, with dark-adapted eyes being significantly slower. A previous study comparing dark- and light-adapted *Leptuca pugilator* fiddler crabs showed that FFF reduced from 50 Hz in a light-adapted state to 32 Hz when dark-adapted (Layne et al., 1997). This is similar to the 73.6 to 34.5 Hz (dark) reduction measured in our experimental animals during daytime (Fig. 3).

There was no difference in the FFF in dark-adapted *G. dampieri* between day and night. This suggests that the temporal summation was operating at maximum efficiency during the experiment at both times. In contrast, there was a clear circadian difference in FFF in light-adapted eyes (15 Hz slower at night). This seems slightly counterintuitive, as we predicted that vision would maintain its fast speed after dusk in the presence of bright light, or even speed up further to counteract any anatomical changes that had started to increase the light-gathering capacity of the eye. There are two possible explanations for vision slowing instead. The first is that the animals have begun to anatomically prepare for dim light and are therefore poorly adapted to the bright light conditions they experience during the experiment. This would disrupt photoreception and interfere with their temporal contrast sensitivity, reducing FFF as a consequence. The second explanation is that the temporal summation is also under circadian control. Just like the size of the rhabdoms, the crabs are not able to control this completely in response to light. For instance, the circadian clock could regulate ion channels in the photoreceptor membrane, possibly with serotonin as in the locust (Cuttle et al., 1995). In *Limulus*, efferent optic nerve impulses are sent to the photoreceptors at night to increase the signal gain (response per photon) to improve dim light vision (Barlow, 1983; Barlow and Kaplan, 1977; Barlow et al., 1987; Kaplan et al., 1990). This works by filtering away fast ‘noisy’ quantum bumps by altering the ionic conductance of the photoreceptor cell membranes, amplifying slower signals and improving photon absorption (Barlow et al., 1987; Honkanen et al., 2017). Assuming this phenomenon occurs in fiddler crabs, this gain increase appears to be only partially inhibited by the continued exposure to bright light before and during the experiment.

Slowed vision in dim light also presents an energy saving opportunity. The metabolic cost of producing much slower signal responses is reduced (Laughlin et al., 1998; Laughlin and Weckström, 1993). These savings could be important given the substantial energetic investment required for production of large volumes of photoreceptor membrane every dusk to widen the rhabdom. These specific cost–benefit offsets in compound eyes warrant further examination.

Spatial integration is another potential strategy for increasing sensitivity of the visual system. It could contribute to the increase in sensitivity for dark-adapted animals during the day, but not the fast decrease in FFF in the dark. This was not investigated in this study but it could be tested in future by comparing spatial resolving power in light- and dark-adapted crabs using the pattern electroretinography (PERG) technique (Ogawa et al., 2019; Porciatti, 2007). Evidence for spatial summation could also be obtained via identification of lateral branching dendrites from the laminar monopolar cells or higher order interneurons, which reach over to collect and pool visual signals from neighbouring ommatidia, at the expense of spatial resolution (Honkanen et al., 2017; Ribi, 1977; Warrant, 2017, 1999). So far, there is no such evidence to support spatial integration in a brachyuran crab.

### Rates of dark adaptation

Experiment 3 showed that the time course and extent of dark adaptation was different at night and during the day. At first, sensitivity was similar when the animals were fully light-adapted. However, the initial adaptation to dim light happened much faster at night, the responses increasing to 5 times larger than the day level after just 12 min. This difference was likely due to circadian metabolic organelle preparations in the photoreceptors, ahead of the experiment. Organelles such as opsin-filled vesicles are produced in late afternoon, filling the soma in anticipation of night so that the rhabdoms can begin widening as soon as the evening light fades (Stowe, 1980; Matsushita and Arikawa, 1997; Matsushita et al., 1999). Although dark adaptation at night reached an initial peak after 12 min, it took approximately twice as long during the day. Whether this is a real difference in the rate of the adaptation process during the day and the night is unclear because of the subsequent unexpected oscillations in response amplitudes that are evident after 12 min (Fig. 4). The oscillations are likely to be an interaction with the test light, and in some individuals the amplitude of these was extreme. The higher final sensitivity of the animals during the control test suggests that the stimulation lights we used had interfered with the adaptation process. We believe that the animals' initially insensitive eyes underwent dark adaptation, only to have a light-adaptation response later (∼12 min) when their newly sensitive eyes were able to detect it well, appearing bright. When light adaptation had ensued for some time, the eye became, once again, insensitive to the test light. A more careful experimental design that only tests each animal once at a predetermined time point will be necessary to resolve this issue.

The conspicuous oscillations caused by the test light demonstrates the strong disruption that even dim intermittent light exposure can have on the process of dark adaptation. The great effect it had on individual responses is something to consider when working with animals that are assumed to be dark-adapted, where utmost caution is important to prevent accidental light exposure prior to or during experiments. It has additional ecological implications for fiddler crabs that live in areas of artificial light pollution at night, such as *A. tangeri*, which are active after sunset (Altevogt and Hagen, 1964; Hagen, 1962; Wolfrath, 1993; Brodrick et al., 2021). Although less is known about how light pollution affects the eye physiology of nocturnal animals, many are known to experience disruptions in visual perception and behaviour (e.g. Underwood et al., 2017; Gaston et al., 2013).

### Sex differences

Overall, ERG response amplitudes in the females were slightly larger than the males when presented with the same range of stimulus intensities in experiment 1. Mean carapace width in the males was only 1 mm larger than females (*n*=4). Male *G. vomeris* tend to have slightly larger eyes than equivalent sized females (Smolka et al., 2011). As larger animals and compound eyes are often correlated with larger facet lenses (Rutowski, 2000; Spaethe and Chittka, 2003; Zagorski and Merry, 2014), this would suggest that, if anything, the ommatidia in males would have slightly greater light-gathering power (Snyder, 1979; Warrant and Nilsson, 1998), leading to better absolute sensitivity; however, the results suggest the opposite. Sex differences in fiddler crab visual abilities were not found in experiments 2 and 3 and are not otherwise known, so this particular result may be coincidental.

### Summary

**Fig. 6.**
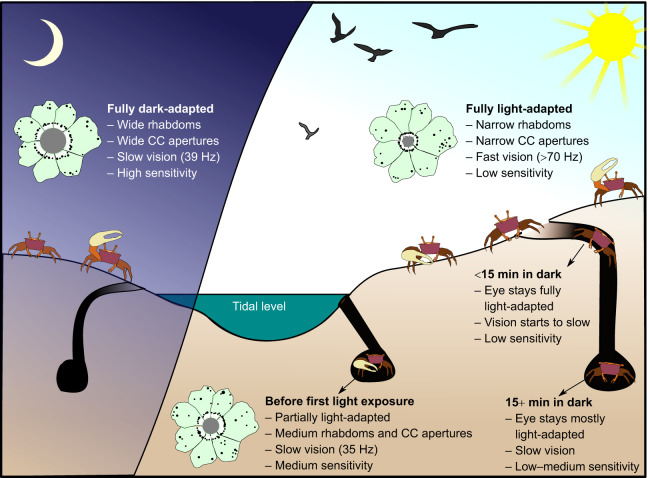
**Summary of the major visual adaptations experienced by fiddler crabs from night to day.** Fiddler crabs must cope with extreme changes in brightness, in and out of their burrows and between night (left) and day (right). At night, dark-adaptation processes in the eye allow it to effectively detect small contrasts in dimly lit scenes, allowing extended foraging time. During daytime, the crabs' ommatidia have small crystalline cone (CC) apertures and narrow rhabdoms, which together with short integration times provide fast vision appropriate for the very bright light conditions on the mudflat. In anticipation of dusk, the ommatidia increase their size and apertures. When light levels drop, the eye slows down and increases sensitivity. Diagrammatic ommatidia in cross-section show relative sizes of *Afruca tangeri* rhabdoms (grey) and unchanging pigment granule distributions (black dots) in the cytoplasm of photoreceptors (pale green), scaled using data from Brodrick et al. (2021).

Insights from our ERG experiments combined with anatomical data from *A. tangeri* (Brodrick et al., 2021) provide consistent evidence that fiddler crabs are not only highly capable of adapting their visual system to enhance vision in dim light, but also that they do so very effectively (Fig. 6 summarises the night-to-day visual adjustments). Our discussions were made assuming the eyes of *G. dampieri* undergo equivalent adaptive anatomical changes as reported in *A. tangeri* and other crabs with similar circadian clocks (Brodrick et al., 2021; Leggett and Stavenga, 1981; Nässel and Waterman, 1979; Rosenberg and Langer, 2001), and initial histological examinations of *G. dampieri* eyes support this (Fig. 5). Time of day and prior light adaptation both had a strong effect on ERG responses and, therefore, eye sensitivity. The circadian difference in adaptation strategies would serve to optimise vision for bright sunlight on mudflats with intermittent burrow use, whilst promoting nocturnal activity during or after sunset. Jocelyn Crane, who spent many years studying fiddler crabs across the globe, reported nocturnal activity for many species (Crane, 1975), which is often associated with courtship behaviours using acoustic signalling, exclusive to this time of day (Salmon and Atsaides, 1968). However, it remains to be properly established whether *G. dampieri* are routinely nocturnally active in their natural habitat. It is likely though, as video surveillance of our artificial mudflat facility shows that the crabs are active outside their burrows regularly at night, even underwater during their ‘high tide’ period. In addition to slow anatomical adaptations, we demonstrate that temporal summation is employed as a dark-adaptation strategy in *G. dampieri* during both day and night. This is likely to operate on a faster time scale of several minutes and are reversible.
